# Supplementary material for: Smooth Interpolating Curves with Local Control and Monotone Alternating Curvature
Source: Comput Graph Forum. 2022 Oct 6;41(5):25–38. doi: 10.1111/cgf.14600 (PMC9827861; doi:10.1111/cgf.14600)
Supplement: Supplementary file 1 — Supplement Material [file CGF-41-25-s001.zip › Local-Smooth-Interpolating-MonoCurvature/extern/clothoids/submodules/Utils/docs/api-cpp/struct_a00087.html]

Template Struct basic\_data — Utils v1.2

### Navigation

- index
- toc
- next
- previous
- Utils »
- C++ API »
- Template Struct basic\_data

# Template Struct basic\_data¶

- Defined in File format.cc

## Struct Documentation¶

template<typename T = void> struct detail::basic\_data¶
:   Public Static Attributes

    static FMT\_API constexpr const char digits [100][2] = {{'0', '0'}, {'0', '1'}, {'0', '2'}, {'0', '3'}, {'0', '4'}, {'0', '5'},{'0', '6'}, {'0', '7'}, {'0', '8'}, {'0', '9'}, {'1', '0'}, {'1', '1'},{'1', '2'}, {'1', '3'}, {'1', '4'}, {'1', '5'}, {'1', '6'}, {'1', '7'},{'1', '8'}, {'1', '9'}, {'2', '0'}, {'2', '1'}, {'2', '2'}, {'2', '3'},{'2', '4'}, {'2', '5'}, {'2', '6'}, {'2', '7'}, {'2', '8'}, {'2', '9'},{'3', '0'}, {'3', '1'}, {'3', '2'}, {'3', '3'}, {'3', '4'}, {'3', '5'},{'3', '6'}, {'3', '7'}, {'3', '8'}, {'3', '9'}, {'4', '0'}, {'4', '1'},{'4', '2'}, {'4', '3'}, {'4', '4'}, {'4', '5'}, {'4', '6'}, {'4', '7'},{'4', '8'}, {'4', '9'}, {'5', '0'}, {'5', '1'}, {'5', '2'}, {'5', '3'},{'5', '4'}, {'5', '5'}, {'5', '6'}, {'5', '7'}, {'5', '8'}, {'5', '9'},{'6', '0'}, {'6', '1'}, {'6', '2'}, {'6', '3'}, {'6', '4'}, {'6', '5'},{'6', '6'}, {'6', '7'}, {'6', '8'}, {'6', '9'}, {'7', '0'}, {'7', '1'},{'7', '2'}, {'7', '3'}, {'7', '4'}, {'7', '5'}, {'7', '6'}, {'7', '7'},{'7', '8'}, {'7', '9'}, {'8', '0'}, {'8', '1'}, {'8', '2'}, {'8', '3'},{'8', '4'}, {'8', '5'}, {'8', '6'}, {'8', '7'}, {'8', '8'}, {'8', '9'},{'9', '0'}, {'9', '1'}, {'9', '2'}, {'9', '3'}, {'9', '4'}, {'9', '5'},{'9', '6'}, {'9', '7'}, {'9', '8'}, {'9', '9'}}

    static FMT\_API constexpr const char hex\_digits []  = "0123456789abcdef"

    static FMT\_API constexpr const char signs [4]  = {0, '-', '+', ' '}

    static FMT\_API constexpr const char left\_padding\_shifts [5] = {31, 31, 0, 1,0}

    static FMT\_API constexpr const char right\_padding\_shifts [5] = {0, 31, 0, 1,0}

    static FMT\_API constexpr const unsigned prefixes [4] = {0, 0, 0x1000000u | '+',0x1000000u | ' '}

### Quick search

### Table of Contents

- rang - Colors for your Terminal
- Zstr
- C++ API

«
hide menu

menu
sidebar
»

### Navigation

- index
- toc
- next
- previous
- Utils »
- C++ API »
- Template Struct basic\_data

© Copyright 2021, Enrico Bertolazzi.
Created using Sphinx 4.2.0.
